# Fitness Trade-Offs Resulting from Bacteriophage Resistance Potentiate Synergistic Antibacterial Strategies

**DOI:** 10.1128/IAI.00926-19

**Published:** 2020-06-22

**Authors:** Mihnea R. Mangalea, Breck A. Duerkop

**Affiliations:** a Department of Immunology and Microbiology, University of Colorado School of Medicine, Aurora, Colorado, USA; University of California, Santa Cruz

**Keywords:** antibiotic resistance, bacterial fitness, bacterial infection, bacteriophages, colonization, phage receptor

## Abstract

Bacteria that cause life-threatening infections in humans are becoming increasingly difficult to treat. In some instances, this is due to intrinsic and acquired antibiotic resistance, indicating that new therapeutic approaches are needed to combat bacterial pathogens. There is renewed interest in utilizing viruses of bacteria known as bacteriophages (phages) as potential antibacterial therapeutics. However, critics suggest that similar to antibiotics, the development of phage-resistant bacteria will halt clinical phage therapy. Although the emergence of phage-resistant bacteria is likely inevitable, there is a growing body of literature showing that phage selective pressure promotes mutations in bacteria that allow them to subvert phage infection, but with a cost to their fitness. Such fitness trade-offs include reduced virulence, resensitization to antibiotics, and colonization defects. Resistance to phage nucleic acid entry, primarily via cell surface modifications, compromises bacterial fitness during antibiotic and host immune system pressure. In this minireview, we explore the mechanisms behind phage resistance in bacterial pathogens and the physiological consequences of acquiring phage resistance phenotypes. With this knowledge, it may be possible to use phages to alter bacterial populations, making them more tractable to current therapeutic strategies.

## Abstract (precis)

Bacteria that cause life-threatening infections in humans are becoming increasingly difficult to treat. In some instances, this is due to intrinsic and acquired antibiotic resistance, indicating that new therapeutic approaches are needed to combat bacterial pathogens. There is renewed interest in utilizing viruses of bacteria known as bacteriophages (phages) as potential antibacterial therapeutics. However, critics suggest that similar to antibiotics, the development of phage-resistant bacteria will halt clinical phage therapy.

## INTRODUCTION

Bacteriophages (phages) contribute to the evolution of bacterial communities, populations, and genomes by maintaining microbial diversity through coevolutionary mechanisms (1–3). While arms race dynamics have been classically observed in laboratory phage-bacterium experiments (4), renewed interest in the fitness costs associated with phage resistance has identified fluctuating selection dynamics as a competing concept for host-parasite dynamics over longer evolutionary periods (2, 5). Lytic phages must first recognize and adsorb to receptors on the bacterial surface, structures in which mutations may prove costly, thereby promoting frequency-dependent selection (5). The lysis of host bacteria following the production of viral progeny can significantly alter bacterial population densities impacting microbial ecosystems. This may not be surprising considering that phages are predicted to outnumber bacteria in the environment (6) and in host-associated microbiotas (7–9), although these phage and bacterial counts are estimates that require further characterization. Phage selection can maintain steady-state bacterial community composition via classic predator-prey dynamics, yet bacteria evolve phage resistance that as a consequence introduces fitness costs and trade-offs in return (10). Evolved and intrinsic bacterial phage resistance drives antagonistic coevolution that can shape phage and bacterial host genomes (11). Bacterium-phage coevolution produces consecutive rounds of mutations whereby bacteria evolve resistance to initial adsorption or infection, followed by phage host range mutations to overcome resistance. In many instances, bacterial resistance to lytic phages comes with fitness costs in heterogeneous populations (12), as well as host colonization defects (13) and dampening of virulence (14). The fitness costs incurred by resistance to one or more phages are grounded in genotypic mutations that produce a spectrum of resistance phenotypes with varyingly poor pleiotropic effects (15). Fixed mutations and transient cell surface modifications promote phage resistance in bacteria during exposure to lytic phages. Phage resistance phenotypes with associated fitness defects could possibly be exploited during phage therapy against overt and opportunistic bacterial pathogens.

The model system for the study of bacterium-phage coevolution is largely based on interactions between Escherichia coli and T-type lytic phages (10, 11, 15, 16). These studies show that phage-resistant E. coli strains are often at a fitness disadvantage to their phage-sensitive relatives, and the magnitude of this trade-off is dependent on the genetic basis of resistance and on the environmental context (17, 18). In complex environments such as soil, the evolution of resistance to phages is more costly than that *in vitro*, and phage selective pressure is a stronger driver of mutation than is competition with bacterial community members (3). Given that evolved phage resistance often involves mutations in bacterial cell surface receptors that are important for cellular stability and function, it is clear why such mutations can be detrimental.

Recently, there has been renewed interest in phage therapies for recalcitrant bacterial infections. Phage therapy in combination with antibiotics is hypothesized to be a potent synergistic attack on target bacteria that impedes the evolution of resistance due to fitness constraints (19). During infection, when colonization and host pressures drive virulence factor production in bacteria, the added pressure of phage predation can reduce virulence as a trade-off for resistance (12). By taking advantage of bacterial resistance, phage therapy can steer pathogenic bacteria toward deleterious surface mutations that allow for more favorable treatment outcomes (20). Combination therapies with phage and antibiotics are clinically promising (21), and phage-antibiotic synergy (22) has been proposed as an innovative therapeutic option for antimicrobial-resistant bacteria (23). Predatory phages can utilize critical bacterial surface molecules that provide either defense from antibiotics via efflux, uptake of nutrients in resource-limited host environments, or general cell wall maintenance. The association between phage receptor molecules and the bacterial response to antibiotic stress is therefore an important consideration for phage therapy.

Understanding the fitness costs associated with bacteriophage resistance is paramount for developing targeted therapies for bacterial infections. Bacterial resistance to lytic phage infection can lead to decreased fitness, and reduced virulence can be a trade-off in biologically relevant situations such as *in planta* colonization (24). Since bacterial resistance to phages may be inescapable, we should consider that phage resistance could be exploited and used against bacteria during antibacterial therapies. In this minireview, we explore this idea through a discussion of phage infection mechanisms, how bacteria subvert phage attack through resistance, and the physiological outcomes of bacterial immunity to phages. Understanding the extent of bacterial fitness defects as a result of phage resistance will be valuable for translational research aimed at developing phages as therapeutics for difficult-to-treat bacterial infections.

## CELL SURFACE-ASSOCIATED MOLECULES AND MACROMOLECULAR STRUCTURES AT THE INTERFACE OF PHAGE-BACTERIUM INTERACTIONS

### Polysaccharides.

To infect bacterial cells, phages must bind to the surface of their susceptible host by accessing cell wall-associated molecules that serve as receptors (25). Polysaccharides, the most abundant extracellular biopolymers produced by bacteria, provide important structural and functional benefits to bacterial cells (26, 27). Exopolysaccharide antigens, covalently attached capsular polysaccharides, and lipopolysaccharides have been described as phage receptors in both Gram-negative and Gram-positive bacteria (28). Early phage-bacterium interaction studies in Escherichia coli revealed the bacterial capsule as a primary receptor for viral spike tip proteins (29). Conversely, the capsule of Staphylococcus aureus can block phage access to cell wall receptors (30). Given the dynamic complexity of bacterial glycan synthesis and phenotypic switching, polysaccharide profiles can determine phage resistance (31, 32) and host ranges (33, 34). The lytic phage strategy of initial reversible recognition via adsorption and irreversible binding to cell surface receptors (35) implicates the glycan cell wall profile as a key determinant of phage infection and resistance.

Interactions with bacterial polysaccharides can be dictated by phage-borne polysaccharide depolymerase enzymes that degrade polysaccharides during phage adsorption, facilitating phage binding to primary receptors on the cell surface (33). Phage tail spike proteins such as glycoside hydrolases and endosialidases that digest bacterial polysaccharides are important for initial binding and subsequent infection (36). E. coli K1-specific phages have capsule-specific tail spike proteins with endosialidase activity that drill through the polysialic acid capsule (36–39). Host specificity for K1 phages is determined by these endosialidases and can specify phage host range (40, 41). For example, the similarity of cell wall polysialic acid linkages of E. coli K1 and *Salmonella* spp. allows for indiscriminant infection by the enteric phages phi92 (34) and SP6 (41) due to polysaccharide-degrading tail spike proteins. Additionally, exopolysaccharides produced by Acinetobacter baumannii are receptors for phages that encode strain-specific tail spike polysaccharide depolymerase enzymes (42). For detailed examination of phage-encoded polysaccharide depolymerases that bind and degrade bacterial glycans, see the study by Latka et al. (43). Given the potential for broad-host-range depolymerases uncoupled from phages, these enzymes are being explored as therapeutic antibacterials against encapsulated bacterial pathogens (44–47).

### LPS.

Lipopolysaccharide (LPS), a primary component of the Gram-negative bacterial cell wall, is another extracellular glycan linked to phage adsorption. The E. coli phage T4 uses LPS as a primary receptor, whereby saccharide distribution and arrangement at the cell surface determine infectivity on a strain-specific basis (48, 49). Recent research on T4-E. coli interactions describes divergent adsorption strategies to bind directly to LPS with or without help from the outer membrane protein OmpC (50). Similarly, *Salmonella* phages specifically recognize the O-antigen portion of LPS via carbohydrate-binding domains on tail spike proteins (51). Given the stratification of LPS into distinct oligosaccharide and core components, it may be necessary for phages to bind to the primary O antigen before accessing secondary receptors (52). Indeed, E. coli phage G7C was recently shown to deacetylate O-antigen polysaccharide during adsorption, and E. coli requires this glycan substrate for successful infection (53). LPS and O-antigen binding kinetics can vary due to different interaction strategies employed by phages that infect LPS-producing bacteria, and the use of LPS-modified phage-resistant mutants has been useful for characterizing new phage isolates (54). Phage-driven selection of O-antigen serotypes has undoubtedly led to diverse LPS varieties.

### Teichoic acids.

In Gram-positive bacteria, teichoic acids amid a peptidoglycan layer can serve as phage receptors (55–57). Lipoteichoic acids of Lactobacillus delbrueckii have been proposed to function as primary phage receptors (58). Additionally, wall teichoic acids, which are highly abundant in the cell wall of S. aureus, are proposed to be directly bound of the phi11 baseplate protein through carbohydrate binding (59). Recent structural and bioinformatic analyses have revealed a highly conserved carbohydrate binding module in lactococcal tail proteins that underscores the importance of glycan binding by phages prior to infection (60). Thus, it is clear that the available sugar moieties of the bacterial cell wall play important roles in phage-bacterium interactions. This is evident considering that wall teichoic acids are often decorated with glycosyl groups that promote phage adsorption (61, 62). For a more detailed description of cell wall receptors targeted by Gram-positive phages, readers are directed to a recent review of this topic (63).

### Cell wall polysaccharides.

In lactic acid bacteria, cell wall polysaccharides (CWPS) are involved in important physiological fermentative processes (64). Recognition of specific saccharide motifs on Lactococcus lactis pellicles is required for adsorption of lactococcal phages to their hosts in a strain-dependent manner (65). Diversity among lactococcal cell wall polysaccharides has led to the development of narrow-host-range phages based on pellicle genotype (66, 67). The complexity of glycan cell surface phage receptors is important for subtype categorization of lactococcal strains based on phage sensitivity profiling (33). The host ranges of Streptococcus thermophilus phages have also been linked to host exopolysaccharide gene operon content (68, 69). In Streptococcus mutans, a rhamnose-glucose polysaccharide produced by the *rgp* biosynthetic operon is important for phage adsorption (70).

### Enterococcal polysaccharide antigen.

In enterococci, the enterococcal polysaccharide antigen (Epa) has been proposed as a phage receptor necessary for adsorption (71). The *epa* cluster, consisting of 18 core genes and strain-specific variable genes, encodes enzymes that produce a rhamnopolysaccharide that is highly conserved in Enterococcus faecalis and Enterococcus faecium (72). The modification of the Epa polysaccharide by glycosyltransferases is required for successful phage adsorption, and recent work indicates that Epa may function as an initial attachment factor for the localization of phages to a secondary receptor (9, 71, 73). Mutations in either core or variable *epa* genes in E. faecalis reduce phage adsorption and significantly alter phage infectivity (9, 73, 74). Thus, much in the same way that CWPS production in lactococci mediates phage host ranges, Epa is an important extracellular mediator of enterococcal phage adsorption and a host range determinant.

### Outer membrane proteins.

Beyond the complexity of phages having to adapt to rapidly changing bacterial surface glycans, a variety of outer membrane proteins also mediate phage-bacterium interactions. A disparity exists between phages that prefer protein and those that prefer polysaccharide receptors, with some overlap, although pathogens that produce multiple LPS moieties, such as Pseudomonas aeruginosa, attract sugar-binding phages (28). Alongside LPS, early studies of coliphage adsorption to E. coli K-12 identified the maltose transporter LamB (75), as well as OmpC (49), an outer membrane porin, as the primary receptors during phage infection. OmpC recognition by phages appears to be conserved in other enteric bacteria, such as the nosocomial pathogen Klebsiella pneumoniae (76). In addition, the outer membrane protein OmpF is also an important phage receptor of enteric bacteria (77). Interestingly, some phages can adapt to use any of the three above-mentioned surface proteins for their adsorption (78), which is further specified by host-specific LPS modifications (50, 79). Given the relevance of OmpF and OmpC as membrane porins for the incorporation of β-lactam antibiotics (80), their moonlighting as phage receptors becomes an important consideration for the selection of therapeutic phages that target enteric bacteria and the potential consequence of phage resistance curtailing antibiotic efficacy.

The iron-siderophore transport protein FhuA (formerly TonA) has been identified as a receptor for several enteric phages (81). TonB, required for the energy-dependent uptake of low-concentration substrates like siderophore transport by FhuA, is also a phage receptor in various enteric bacteria (82). Additionally, the vitamin B_12_/cobalamin outer membrane porin BtuB is a receptor for multiple T5-like phages (83, 84). TolC, the outer membrane component of the multidrug resistance efflux pump AcrAB-TolC, is a receptor that does not constrain the phage host range to serovar specificity in Salmonella enterica (85). Likewise, TolA, part of the Tol system of membrane proteins that imports macromolecules and links the inner and outer membranes of E. coli, acts as a filamentous phage receptor (86, 87). It is notable that the majority of outer membrane proteins described as phage receptors in Gram-negative bacteria are also important for pathogen survival in hosts.

### Phage infection protein.

Much of what is currently known about membrane proteins that function as phage receptors in Gram-positive bacteria stems from the identification of the lactococcal phage infection protein (PIP) (88). In conjunction with CWPS, PIP facilitates irreversible adsorption to the membrane by lactococcal phages (89). Similar PIP-like proteins have been characterized as phage receptors in pathogenic and nonpathogenic Bacillus species, with GamR in B. anthracis (90) and YueB in B. subtilis (91). Like the interaction between CWPS and PIP in L. lactis, the B. subtilis-specific phage SPP1 first binds reversibly to glucosylated wall teichoic acids before irreversibly binding to YueB (92). In E. faecalis, an orthologous protein, PIP_EF_, promotes infection of enterococcal phages and dictates phage tropism through a variable region extracellular domain (93). PIP_EF_ is predicted to function in small-molecule or protein transport based on homology to the type VIIb secretion system protein EsaA in S. aureus (93), a membrane protein with six transmembrane helix domains conserved in Staphylococcus and Listeria spp. (94). The use of conserved bacterial outer membrane proteins that are functionally important to their host’s survival ensures consistent and accessible options for phage adsorption.

### Flagella.

Flagella enable bacterial motility through a variety of environmental and host-associated substrates. Perhaps also due to their functional ubiquity, these bacterial appendages serve as receptors to a variety of phages. Phages can attach to the flagellar filaments of E. coli and adsorb to the base of the flagella during infection (95). More recently, a phage targeting Salmonella enterica serovar Typhimurium was shown to be flagellotropic but only infectious when flagella rotate counterclockwise (CCW) (96). Flagellotropic phage χ also depends on CCW flagellar rotation in addition to flagellar filament surface groove structure for successful infection of E. coli and S. enterica serovar Typhimurium, termed the “nut-and-bolt” strategy (97). Phages using S. enterica serovar Typhimurium flagella as a receptor can be further differentiated into subgroups that target single or multiple flagellar proteins and subunits (98). The χ-like phage of S. enterica serovar Typhi uses flagella as the primary receptor for infection (99); however, Δ*fliC* and Δ*fljB* mutants that lack the flagellar filament can still be infected with reduced efficiency. This observation is consistent with a model of flagellotropic infection of Agrobacterium spp. (100), whereby infection with lytic phages requires contact with LPS and is facilitated by flagellar rotation. Flagella are also important for initial phage adhesion to Caulobacter crescentus (101), in which attachment is also dependent on CCW rotation that enables phage contact with pilus portals as final receptors at the bacterial pole.

### Pili.

Pilus structures are commonly utilized as receptors for a variety of phages targeting bacterial pathogens. Phages recognize and infect using type IV pili (T4P) in P. aeruginosa (102, 103), as well as the tip of the F conjugative pilus (104) in conjunction with the TolQRA complex in E. coli (105). T4P are on par with flagella regarding their functional ubiquity among bacteria; the pilus molecular machine that contributes to motility and adherence is a key virulence factor for opportunistic human pathogens like P. aeruginosa (106). Studies of pilus-specific phages recently identified two lytic phages that can infect both P. aeruginosa and Stenotrophomonas maltophilia via T4P as the surface receptors (107). The utility of T4P as phage receptors therefore provides broad-host-range specificity (108), an advantage in phage therapy applications. In Vibrio cholerae, the toxin-coregulated pilus, a T4P family structure that mediates host colonization, is the primary receptor for the filamentous phage CTXϕ (109). Targeting virulence factors such as T4P via phage therapy has been proposed as an effective antivirulence method that could clear multiple-species infections while also selecting for less-virulent resistant bacteria (107). Thus, understanding phage-pilus interactions in the context of bacterial fitness and virulence in the host is an important consideration for many bacterial pathogens.

### Summarizing thoughts.

**FIG 1:**
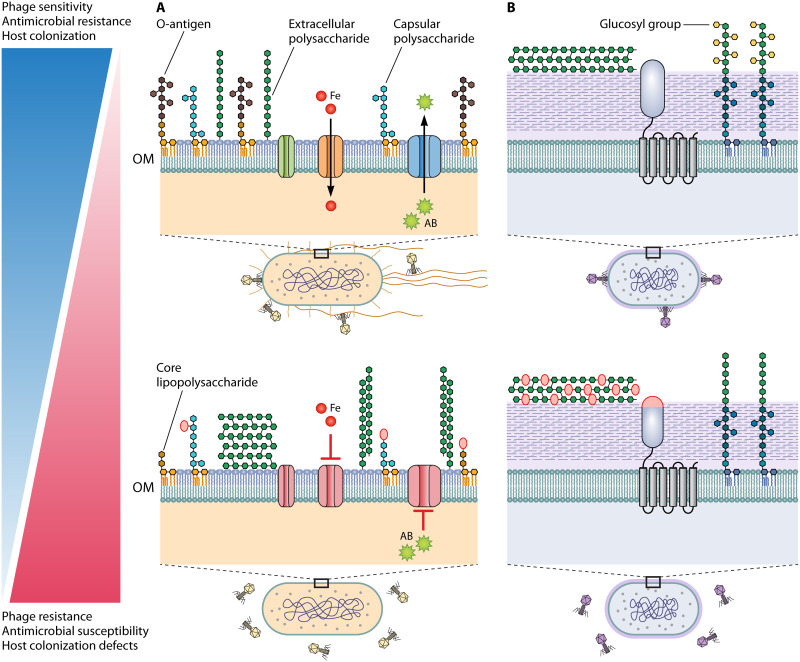
Bacterial cell surface mutations and modifications produce fitness trade-offs between bacteriophage resistance, antimicrobial susceptibility, and host colonization. (A and B) Model illustrations of prototypical Gram-negative (A) and Gram-positive (B) cell walls and outer membrane (OM) structures that serve as phage receptors. (A) Top, a flagellated and piliated Gram-negative rod is depicted with functional efflux (blue), siderophore (orange), and porin (green) complexes, amid a diverse array of lipopolysaccharide, capsular polysaccharide, and extracellular polysaccharide structures. Bottom, the Gram-negative rod is without functional OM protein complexes, increased exopolysaccharide production, and a truncated and mutated lipopolysaccharide profile. (B) Top, a Gram-positive bacterium is depicted with extracellular polysaccharide antigen, the PIP membrane protein (gray), and glucosylated wall teichoic acids. Bottom, the same Gram-positive bacterium displays modified glycans in the extracellular polysaccharide antigen, mutated PIP variable region, and undecorated wall teichoic acids. Sugar moiety modifications are depicted with red ovals, and genetic mutations affecting proteins are also depicted in red. (A and B) The adjacent gradient illustrates the fitness trade-offs associated with sensitivity (top) and resistance (bottom) to phages, traits which can be inversely correlated with antimicrobial sensitivity and host colonization potential.

The bacterial cell surface is a rich landscape of receptor molecules for phage adsorption and infection. Because phages are nonmotile and depend on localized chance interactions with their hosts, their range and specificity are dictated by their adaptation to host cell surfaces. The first line of bacterial defense against phage infection lies in bacterial surface molecules. Surface polysaccharides, integral membrane proteins, and appendages that are externally exposed on bacterial cells are also antigens, virulence factors, and essential transporters of nutrient cofactors and efflux. Modification and/or mutation of these cellular components is likely to incur fitness costs, including decreased resistance to environmental pressures and virulence reduction (Fig. 1).

## BACTERIAL SURFACE-BASED MUTATIONS MEDIATE PHAGE RESISTANCE

The omnipresent threat of phage infection has led to the evolution of several intrinsic bacterial defense systems, including CRISPR-Cas proteins and restriction-modification systems that protect bacteria from invasive foreign DNA (110). Abortive infection systems (111) facilitate the self-destruction of infected bacterial cells to reduce the phage infection burden within a population. These resistance mechanisms are effective but are not infallible and can be overcome by phage infectious dose and host range mutations that allow phages to subvert bacterial immunity after their genomes are inside cells (112). Mechanisms that block initial phage adsorption to the cell and prohibit infection are selected during evolution of bacterial resistance, in spite of fitness defects resulting in bacterial competitive disadvantages (113). Much in the same way antibiotic resistance can evolve in bacteria, resistance to phages can develop via individual mutations to phage receptors, the presence of competitive inhibitors of phage receptors, or restriction of receptor access with polysaccharides. In this section, we describe these bacterial self-defense methods in the context of lytic phage coevolution.

### Outer membrane protein mutations.

Spontaneous mutations can arise in outer membrane proteins that are required for initial phage adsorption to bacteria. This is especially true for phages with narrow host ranges that attach to specific bacterial proteins. An example is phage λ and the protein LamB in E. coli (114). In a formative coevolution experiment analyzing this relationship, λ-sensitive E. coli repeatedly developed mutations in the *malT* gene that regulates *lamB* expression, resulting in λ becoming fixed in the bacterial population after 8 days (115). The induction of LamB in a subpopulation of E. coli Δ*malT* mutants could maintain λ at low levels before successive mutations allowed the use of OmpF as an alternate receptor (115). The evolution of phage λ when cultured with E. coli presenting either LamB or OmpF, but not both proteins, shows that divergent phage populations arise with unambiguous receptor preferences (116). Thus, mutations that affect bacterial surface proteins ultimately drive phage identification and host ranges, although surface polysaccharide composition further complicates this paradigm. In Yersinia pestis, mutations in the outer membrane proteins OmpF and Ail result in defective phage receptors but do not confer complete resistance to infection due to the involvement of LPS as an additional host factor for adsorption (117). Although mutations in surface proteins can be sufficient for bacterial resistance, as measured by zero phage adsorption potential (118), multiple linked mutations may also be necessary for full phage resistance (119). Indeed, phage resistance in Y. pestis is dependent on one or multiple mutations in genes encoding core LPS components or glycosyl modifications to the LPS core (Fig. 1) (120).

### Surface glycome modifications.

In addition to mutations in phage receptors that either reduce their abundance or alter specificity, an effective phage evasion strategy lies in non-mutation-derived modification. Given that cell wall polysaccharides such as LPS often mediate phage infection in conjunction with membrane-associated proteins (77), their modification can confer transient resistance to phages without mutations in protein-encoding genes. One example of this strategy is evident in S. enterica serovar Typhimurium, in which phase-variable O-antigen glucosylation confers transient phage resistance (121) without modifying the primary receptor protein BtuB (84). In support of this finding, a recent report shows significant downregulation of LPS synthesis in *Salmonella* spp. upon contact with lytic phages, resulting in up to a 90% reduction in total LPS content (122). Phage-resistant P. aeruginosa mutants also produce truncated LPS structures, which determine full or partial phage resistance profiles (123). Glycome modifications therefore provide further fine-tuning of the bacterial surface as it is presented to their predatory phage that complements the more deterministic approach of fixing mutations within a population to overcome phage infection (Fig. 1).

Phase-variable expression of surface polysaccharides is a bacterial resistance strategy to phage infection that generates population heterogeneity without the burden of mutation. Several examples of this strategy have recently been described in host-associated bacterial pathogens that rely on polysaccharide antigens for colonization but are also under phage-driven selective pressure to modify those antigens. The O1 polysaccharide antigen of the intestinal pathogen Vibrio cholerae undergoes phase variation that hinges on two biosynthetic genes, *manA* and *wbeL*, and determines resistance to the O1 antigen-dependent lytic phage ICP1 (124). Given that V. cholerae O1 is a dominant antigen important for host colonization, it is unsurprising that this key virulence factor is a phage target that must be maintained in a host-associated context (124). A similar observation has been described for the foodborne pathogen Campylobacter jejuni, in which phase variable *O*-methylation modifications to the capsular polysaccharide mitigate phage infectivity in the chicken intestine without affecting colonization (125). It is striking that the predicted number of possible capsule variants in C. jejuni can produce over 700 structures based on phase-variable modifications, implying deep complexity in phage-host coevolution (125). Methylation-driven phase-variable resistance to phage has also been described in the opportunistic pathogen Haemophilus influenzae, whereby the lack of Dam methylation at the *lic2A* locus necessary for lipooligosaccharide (LOS) production drives phase variation and phage resistance (126). Dam methylation also drives phage resistance in S. enterica via the phase-variable *opvAB* operon, which prevents normal LPS O-antigen production required for phage infectivity (127). Variation of pathogenic bacterial cell surface profiles under selective pressure from phage is not limited to polysaccharides. The cell wall protein of the opportunistic pathogen Clostridioides difficile, CwpV, undergoes phase-variable production that protects against phage DNA ejection (128). These phase-variable antiphage systems allow pathogens to establish a strategic middle ground for balancing the demands imposed by both phage predation pressures and host colonization requirements.

### Mucoidy.

A mechanism offering partial resistance from phage predation involves phenotypic mucoid variants that arise via augmented exopolysaccharide production. Mucoidy has classically been described in *Pseudomonas* spp. that produce alginate in response to environmental pressures (129). Coculture with lytic phages promotes a mucoid phenotype conversion in Pseudomonas fluorescens, although P. fluorescens remains partly sensitive to phi2 infection (130). This transient protection from phage predation is considered a partial resistance mechanism to subvert infection by phages that target multiple receptors, and it contrasts the deterministic nature of envelope resistance whereby mutations that modify a single receptor preclude infection by phages (131). In E. coli, mutations in the *rsc* gene cluster involved in exopolysaccharide production were shown to provide phage resistance via mucoidy; however, these mutations were unstable, and bacteria reverted to a nonmucoid phenotype in phage-free cultures, suggesting a fitness cost associated with mucoidy in the context of bacterial competition (132). The instability of mucoidy in environments with fluctuating phage populations can help support phages within a population of bacteria that can revert back to a nonmucoid phenotype (133). Regardless, phage-bacterium evolution models show that mucoidy is a more likely outcome than are fixed cell wall mutations that promote resistance in E. coli (132, 133), suggesting that fixed mutations are more costly.

Considering that natural communities of bacteria are more often in a biofilm rather than a planktonic state, reversible mutations in biofilm-determining loci may be more favorable than all-or-nothing surface receptor modification/mutation. In support of this theory, a recent analysis of phage-biofilm simulations revealed that biofilm heterogeneity supports the coexistence of both phage-resistant and -susceptible bacterial populations (134). In complex environments where multiple phages would be targeting composite bacterial communities, the cost of phage resistance is higher than infection of a single-target population (135); thus, convergent selective pressures can produce multiple mutant phenotypes that arise from equally diverse resistance and immunity strategies. It is important to emphasize that fitness costs to phage resistance are context dependent, as illustrated in a study of plant-associated Pseudomonas syringae that does not incur the same fitness costs *in vitro* (24). This context-dependent disparity is further illustrated by recent work exploring phage resistance evolution, which demonstrates P. syringae phage resistance *in vitro* but not *in planta* (136).

### Summarizing thoughts.

Through spontaneous mutations in outer membrane proteins, modifications to surface polysaccharides, and the conversion to mucoidy phenotypes, bacteria can become resistant to phage predation. Bacterial resistance, resulting in cell surface modifications that preclude phage nucleic acid entry, can lead to fitness defects in host-associated contexts. In addition to single mutations, bacterial resistance to phages can arise through phase-variable glycome modifications that may affect bacterial competitive fitness. Phage-mediated selection of resistant mutants in natural environments has important implications for bacterial pathogenicity reduction and antibiotic intervention strategies.

## PHAGE RESISTANCE FACILITATES ANTIBIOTIC AND HOST IMMUNE SYNERGIES AGAINST BACTERIAL INFECTIONS

The mechanisms of phage resistance discussed so far describe an array of bacterial modifications that impact physiology and consequently lead to fitness defects (Fig. 1). These fitness defects provide opportunities for exploiting bacterial resistance to phages during antimicrobial therapy. Descriptions of reduced virulence and defects in host colonization in response to phage predation have championed the idea of using phages to resensitize bacteria to antibiotics and increase their clearance from host tissues via immune cell targeting.

### Phage-antibiotic combination therapy.

For bacteria with actual or anticipated antibiotic resistance, phage therapy is attractive due to selective pressures and resistance costs that resensitize bacteria to drug treatments (137). One example is E. faecalis, an opportunistic pathogen with emerging resistance to last-resort antibiotics such as vancomycin and daptomycin. Spontaneous mutations in the Epa exopolysaccharide biosynthesis genes provide resistance to lytic phage infection while incurring sensitivity to cell wall- and cell membrane-targeting antibiotics (9, 73). Point mutations in *epaR*, a putative glycosyltransferase and part of the core Epa synthesis gene cluster, prevent phage adsorption and increase susceptibility to daptomycin (73), presumably by altering cell membrane physiology. Likewise, spontaneous mutations in *epaX*, a predicted glycosyltransferase gene in the variable region of the Epa cluster, also provide resistance to phage adsorption (74) but disrupt the ability of E. faecalis to translocate epithelial cell layers or semisolid surfaces (138). Furthermore, our group has shown that additional *epa* variable genes, *epaS* and *epaAC*, are frequently mutated during phage exposure at the cost of enhanced antibiotic sensitivity (9). E. faecalis cells lacking *epaS* are also deficient in intestinal colonization and are more susceptible to vancomycin treatment *in vivo*, indicating that the Epa polysaccharide is important for opportunistic overgrowth in the intestine. Together, these recent discoveries of phage-induced Epa mutations in E. faecalis exemplify key fitness trade-offs for phage resistance and provide support for the idea of synergistic phage-antibiotic combination therapy.

Phages that bind O-antigen chains of LPS structures select for resistant bacteria with distinct surface structures and attenuated virulence. Lytic phages targeting Y. pestis core LPS receptors produced spontaneous phage-resistant LPS mutants whose attenuated virulence in mice reflected truncated LPS lengths (120). Importantly, a majority of these spontaneous LPS mutants developed sensitivity to polymyxin B (120), which indicates that a destabilized outer membrane is a trade-off for phage resistance in these organisms. Listeria monocytogenes was also shown to mutate teichoic acid glycosylation genes in response to phage predation, which led to an attenuation of virulence in an *in vivo* mouse model as well as invasion defects in Caco-2 epithelial and HepG2 hepatocyte cell lines (139). Virulence attenuation as a cost of phage resistance also manifests epigenetically. The phase-variable control of O-antigen chain length in S. enterica triggers transient phage resistance via truncated LPS structures that reduce virulence *in vitro* and *in vivo* (127). Taking into account fitness trade-offs that balance virulence with phage resistance, the use of prophylactic phage cocktails to prevent V. cholerae colonization and infection showed that while phage-resistant V. cholerae isolates are recovered following phage therapy, mutations arise in LPS synthesis genes rendering V. cholerae avirulent in different animal models (140). The use of phages in combination, as cocktails, is also an effective strategy for reducing C. difficile levels in several models (141–143). Multiple-phage combinations targeting C. difficile were effective at reducing the bacterial burden in a hamster model while also mitigating the emergence of resistant bacterial outgrowth (141). C. difficile-specific phages have also been shown to be a useful prophylactic supplement to vancomycin treatment in a wax moth model of infection (142).

Investigation of phage-selected antibiotic resensitization of multidrug-resistant P. aeruginosa revealed that a phage targeting MexAB and MexXY-OprM efflux systems significantly increased sensitivity to clinically relevant antibiotics (144). Phage OMKO1 was efficacious in the treatment of a P. aeruginosa aortic graft infection in conjunction with ceftazidime (145). By exploiting the biological trade-off whereby phage resistance leads to decreased efflux, Chan and colleagues demonstrated the clinical success of OMKO1-ceftazidime therapy based on initial *in vitro* observations (145). Phage-mediated mutation of conserved efflux genes in P. aeruginosa could be a clinically advantageous benefit of phage resistance that may allow for the reintroduction of clinically approved antibiotics for the treatment of multidrug-resistant infections (144). A multidrug-resistant A. baumannii infection has been treated with a personalized phage cocktail in synergy with minocycline, where emergent phage-resistant mutants lacked capsular polysaccharide and became more susceptible to drug treatment (146). This type of molecular synergy has been investigated for *in vitro* biofilms of P. aeruginosa (147) and S. aureus (148). Although the mechanisms that heighten antibiotic sensitivity during phage-biofilm association remain to be described, initial results indicate that phage predation leads to the sensitivity of biofilms to multiple antibiotics (147, 148).

### Phage-immune system synergy.

Similar to phage-antibiotic synergy, increased susceptibility to the host innate immune system is a synergistic feature of phage therapy with great clinical potential. The term “immunophage synergy” has emerged to describe interactions between lytic phages and innate immune defenses (149). Using *in vivo* and *in silico* models of multidrug-resistant Pseudomonas aeruginosa infection, Roach et al. (149) showed that neutrophil activation, and to a lesser extent, MyD88 immune activation, is required for a positive phage therapeutic outcome. The authors suggest that successful phage therapies might depend on the immunocompetency of the host to counter the emergence of phage-resistant bacteria. A previous study of a P. aeruginosa monophage treatment showed a defect in effective phage therapy in neutropenic mice lacking neutrophils (150). These experimental observations are further reinforced by a mathematical model that predicts immunophage synergy in scenarios where neither phages nor the immune system alone is effective at resolving bacterial infections (151). In conjunction with the immunophage synergy theory, a recent study demonstrated that phage-resistant K. pneumoniae mutants were more susceptible to phagocytosis (152). Mutations in glycosyltransferase-encoding genes promoted phage resistance amid deficient capsule synthesis and, subsequently, enhanced *in vivo* predation by macrophages (152). Thus, in addition to phage-antibiotic combination therapies, immunophage synergy is another mechanism by which lytic phages could be used to enhance antibacterial therapies.

The reported synergies between phage therapy and antibiotics or the innate immune system offer promising research avenues to develop combination therapies for clearing bacterial infections. Using phages as a selective pressure to force bacterial mutations that are more susceptible to currently implemented treatment methods, while reducing virulence and colonization fitness, is a strategy that has recently been described as “phage steering” (20). By exploiting the fitness trade-offs experienced by bacteria that evolve resistance to lytic phages through surface modifications, it may be possible to simultaneously reduce bacterial virulence as well as resensitize bacteria to antibiotic or immune killing. In their recent opinion article, Gurney et al. (20) describe the bacterial virulence-associated surface molecules and structures that could be targeted to steer infections toward manageable clinical outcomes. By taking advantage of phage-bacterium coevolutionary biology, it may be possible to reclaim existing clinical strategies for the treatment of bacterial infections, although several important mechanistic questions regarding phage-antibiotic combination therapies remain. Notably, Torres-Barceló and Hochberg (19) point to the importance of discerning precise antibiotic concentrations during combination therapies such that bacterial virulence responses, including quorum sensing and hormesis, are mitigated.

It is also important to consider the polymicrobial nature and host-associated context of bacterial infections that differ dramatically from laboratory conditions. A new study in this domain has shown that although surface modification mutations predominate in bacterial monoculture, the associated fitness trade-offs are exaggerated in a polymicrobial community, leading to CRISPR-mediated adaptation (153). This is an important consideration given that the CRISPR-resistant P. aeruginosa PA14 strain maintained virulence in an *in vivo* infection model (153), suggesting that the mechanism of phage resistance is important for determining infection severity and treatment options. This study highlights the need for relevant infection models that explore phage-bacterium coevolution in the context of polymicrobial interactions that are more representative of real-world scenarios.

## CONCLUDING REMARKS

The fitness trade-offs incurred by phage resistance in pathogenic bacteria offer opportunities for novel intervention strategies for treating recalcitrant infections. By modifying cell surface molecules that serve as phage receptors, bacteria are able to subvert phage infection. Modifications to surface polysaccharides, membrane porins, siderophores, efflux pumps, pili, and flagella can come with substantial fitness defects. Taking advantage of this physiology, phage therapy might be tailored to antibiotic therapy to generate synergy with antibiotics or host innate immune defenses to aid in the clearance of bacterial infections. In addition to these phage-antibiotic and immunophage synergies, there is renewed interest in phage prophylaxis in immunotolerant individuals (149, 154). While phages, antibiotics, or innate immunity may not be singlehandedly sufficient for clearing difficult to treat bacterial infections, it may be possible to push bacterial evolution toward less-fit phenotypes using phage therapy. As a consequence, this phage steering of bacteria may lead to the renewed utility of ineffective antibiotics or aid in immunomodulation (20). Bacterial resistance to phages may therefore provide translational benefits to clinically relevant bacterial infections that no longer respond to conventional therapeutics.
